# Progressive neurologic and somatic disease in a novel mouse model of human mucopolysaccharidosis type IIIC

**DOI:** 10.1242/dmm.025171

**Published:** 2016-09-01

**Authors:** Sara Marcó, Anna Pujol, Carles Roca, Sandra Motas, Albert Ribera, Miguel Garcia, Maria Molas, Pilar Villacampa, Cristian S. Melia, Víctor Sánchez, Xavier Sánchez, Joan Bertolin, Jesús Ruberte, Virginia Haurigot, Fatima Bosch

**Affiliations:** 1 Center of Animal Biotechnology and Gene Therapy, Universitat Autònoma de Barcelona, Bellaterra 08193, Spain; 2 Department of Biochemistry and Molecular Biology, Universitat Autònoma de Barcelona, Bellaterra 08193, Spain; 3 Centro de Investigación Biomédica en Red de Diabetes y Enfermedades Metabólicas Asociadas, Barcelona 08036, Spain; 4 Department of Animal Health and Anatomy, School of Veterinary Medicine, Universitat Autònoma de Barcelona, Bellaterra 08193, Spain

**Keywords:** Lysosomal storage disease, MPSIIIC, HGSNAT, Animal model, Neurodegeneration

## Abstract

Mucopolysaccharidosis type IIIC (MPSIIIC) is a severe lysosomal storage disease caused by deficiency in activity of the transmembrane enzyme heparan-α-glucosaminide N-acetyltransferase (HGSNAT) that catalyses the N-acetylation of α-glucosamine residues of heparan sulfate. Enzyme deficiency causes abnormal substrate accumulation in lysosomes, leading to progressive and severe neurodegeneration, somatic pathology and early death. There is no cure for MPSIIIC, and development of new therapies is challenging because of the unfeasibility of cross-correction. In this study, we generated a new mouse model of MPSIIIC by targeted disruption of the *Hgsnat* gene. Successful targeting left *LacZ* expression under control of the *Hgsnat* promoter, allowing investigation into sites of endogenous expression, which was particularly prominent in the CNS, but was also detectable in peripheral organs. Signs of CNS storage pathology, including glycosaminoglycan accumulation, lysosomal distension, lysosomal dysfunction and neuroinflammation were detected in 2-month-old animals and progressed with age. Glycosaminoglycan accumulation and ultrastructural changes were also observed in most somatic organs, but lysosomal pathology seemed most severe in liver. Furthermore, HGSNAT-deficient mice had altered locomotor and exploratory activity and shortened lifespan. Hence, this animal model recapitulates human MPSIIIC and provides a useful tool for the study of disease physiopathology and the development of new therapeutic approaches.

## Abstract (precis)

**Summary:** A new animal model of the severe neurodegenerative lysosomal disorder mucopolysaccharidosis IIIC recapitulates the human disease, with progressive CNS and somatic lysosomal pathology, and shortened lifespan.

## INTRODUCTION

Mucopolysaccharidosis type III, or Sanfilippo syndrome, encompasses a group of rare lysosomal storage diseases (LSDs) inherited in an autosomal recessive manner and caused by mutations in any of the enzymes specifically involved in the catabolism of the glycosaminoglycan (GAG) heparan sulfate (HS) (Valstar et al., 2008), leading to the accumulation of HS in lysosomes of cells of all tissues and organs. Sanfilippo syndrome is classified into four different subtypes based on the deficient enzyme: MPSIIIA (OMIM#252900) is caused by N-sulfoglucosamine sulfohydrolase (EC 3.10.1.1) deficiency (Kresse and Neufeld, 1972); MPSIIIB (OMIM#252920) by α-N-acetylglucosaminidase (EC 3.2.1.50) deficiency (von Figura and Kresse, 1972); MPSIIIC (OMIM#252930) by heparan-α-glucosaminide N-acetyltransferase (HGSNAT, EC 2.3.1.78) deficiency (Klein et al., 1978); and MPSIIID (OMIM#252940) is the result of N-acetyl-glucosamine-6-sulfatase (EC 3.1.6.14) deficiency (Kresse et al., 1980). MPSIIIA is the most common type of Sanfilippo syndrome in north-west Europe and Australia, and type B is the most frequent type in south-east Europe and Brazil, whereas MPSIIIC and D seem to be much rarer (Coelho et al., 1997; Meikle et al., 1999; Valstar et al., 2008).

All subtypes of Sanfilippo syndrome show similar clinical symptoms, and manifest primarily as progressive neurodegenerative diseases, but MPSIIIC seems to have a milder clinical course although the severity of the disease is highly variable, even within families (Delgadillo et al., 2013; Héron et al., 2011; Ruijter et al., 2008; van de Kamp et al., 1981). After a few months of normal growth, developmental delay and behavioral problems appear as the first signs and symptoms in affected individuals (Héron et al., 2011; Malm and Månsson, 2010; Ruijter et al., 2008). Later on, the disease slowly progresses to neurological deterioration characterized by loss of speech that precedes loss of motor function (Delgadillo et al., 2013; Héron et al., 2011; Malm and Månsson, 2010; Ruijter et al., 2008). During this period, affected individuals show severe behavioral problems with hyperactivity that worsens as a result of sleep disturbances (Delgadillo et al., 2013; Malm and Månsson, 2010; Ruijter et al., 2008). Somatic disease in MPSIIIC, although mild, is characterized by a wide range of clinical manifestations such as recurrent ear-nose-throat infections, diarrhea, mild facial dysmorphisms and visceral organomegaly, such as mild hepatomegaly (Delgadillo et al., 2013; Héron et al., 2011; Ruijter et al., 2008). The largest study on a cohort of individuals with MPSIIIC performed to date was carried out in the Netherlands and showed longer survival for Sanfilippo syndrome subtype C than for subtypes A or B, with death occurring at a median age of 34 years, ranging from 25 to 48 (Ruijter et al., 2008). These data are supported by another study in a smaller cohort from France in which individuals with MPSIIIC lived significantly longer than individuals affected by MPSIIIA or MPSIIIB (Héron et al., 2011). Currently there is no cure for MPSIIIC and existing treatments are aimed solely at controlling the symptoms of the disease in order to improve the quality of life of patients and their families.

HGSNAT is a lysosomal transmembrane enzyme whose function is to N-acetylate the terminal α-glucosamine residues of intralysosomal HS prior to its hydrolysis by α-N-acetylglucosaminidase (Klein et al., 1978). The *HGSNAT* gene is composed of 18 exons and is located on chromosome 8p11.1 (Fan et al., 2006; Hřebícˇek et al., 2006). The cDNA encodes a product of 635 amino acids in length, which contains 11 transmembrane domains, up to five consensus asparagine-linked glycosylation sites, a cleavable N-terminal signal peptide of 30 amino acids, and a short eight-amino acid C-terminal domain facing the cytosol (Fan et al., 2006, 2011; Hřebícˇek et al., 2006). At present, 66 *HGSNAT* mutations have been identified, which include 37 missense mutations, 14 splicing mutations, five small deletions, two gross deletions, five small insertions, one small indel mutation, one gross insertion and/or duplication and one complex rearrangement (https://portal.biobase-international.com/hgmd/pro/gene.php?gene= HGSNAT), and some rare polymorphisms that have no effect on the activity, processing and targeting of the enzyme (Canals et al., 2011; Feldhammer et al., 2009a). Although the spectrum of mutations in individuals with MPSIIIC shows high heterogeneity, some of the identified mutations have high frequency within certain populations, such as p.R344C and p.S518F in the Netherlands (Ruijter et al., 2008), c.525dupT in Portugal (Coutinho et al., 2008; Feldhammer et al., 2009b), c.852–1G4A in southern Italy (Fedele et al., 2007), c.372–2A>G in Spain (Canals et al., 2011) and c.234+1G>A in Spanish and Moroccan individuals with MPSIIIC (Canals et al., 2011; Hřebícˇek et al., 2006; Ruijter et al., 2008), suggesting geographical founder effects. Other mutations, e.g. p.R384*, c.23411G4A, c.49311G4A, p.R344H and p.S541L, show a relatively high incidence among individuals with MPSIIIC from different geographical origins, suggesting a founder effect of an ancient mutation (Fan et al., 2006; Fedele et al., 2007; Feldhammer et al., 2009b; Hřebícˇek et al., 2006). Despite the identification of several MPSIIIC-causing mutations, it has proven difficult to establish a clear genotype-phenotype correlation, except e.g. for mutations p.G262R and p.S539C from two sisters that were associated with an attenuated phenotype (Berger-Plantinga et al., 2004; Ruijter et al., 2008). In any case, the vast majority of individuals with MPSIIIC are affected by at least one missense mutation in or adjacent to transmembrane domains of HGSNAT, interfering with the proper folding of the enzyme (Feldhammer et al., 2009a,b).

Several naturally occurring or engineered small and large animal models of Sanfilippo syndrome have been described in the past few years (Bhaumik et al., 1999; Ellinwood et al., 2003; Fischer et al., 1998; Li et al., 1999; Thompson et al., 1992; Yogalingam et al., 2002), including a rodent model of MPSIIIC (Martins et al., 2015). These animals have proven invaluable to study the ethiopathology of MPSIII, and have been used to establish proof-of-concept for the development of new therapeutic approaches (Crawley et al., 2011; Duncan et al., 2015; Ellinwood et al., 2011; Fu et al., 2011; Haurigot et al., 2013; Langford-Smith et al., 2012; Murrey et al., 2014; Ribera et al., 2015; Sorrentino et al., 2013). Herein, we report a novel murine model of MPSIIIC that recapitulates the human disease at biochemical, histological and functional level, with disease affecting not only the brain but also somatic organs.

## RESULTS

### Generation of *Hgsnat* knockout mouse to develop an animal model for MPSIIIC

**Fig. 1.**
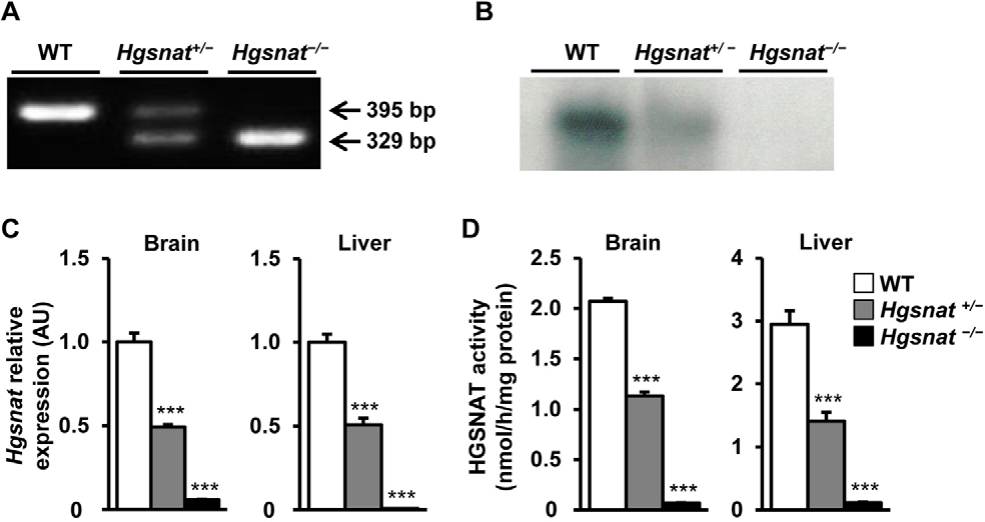
**Generation of *Hgsnat* knockout mouse.** (A) Genotyping of wild-type (WT), heterozygous (*Hgsnat^+/−^*) and homozygous (*Hgsnat^−/−^*) mice was performed by PCR analysis of genomic DNA. The location within the gene locus of the primers used is indicated in Fig. S1. The bands of 395 and 329 bp correspond to WT and mutant alleles, respectively. (B) Representative northern blot performed on total RNA extracted from the kidney of WT, *Hgsnat^+/−^* and *Hgsnat^−/−^* male mice and hybridized with a probe specific for *Hgsnat* mRNA. (C) *Hgsnat* mRNA expression analyzed by qRT-PCR in brain and liver of the same cohorts with primers and probes as listed in Materials and Methods; *n*=4 animals/group. AU, arbitrary units. (D) HGSNAT activity in brain and liver of the same cohorts; *n*=5-7 animals/group. Results are shown as mean±s.e.m.; ***P<0.001 versus WT by one-way ANOVA followed by Dunnett post-test.

Embryonic stem cells (ESCs) with targeted disruption of the *Hgsnat* gene were obtained and microinjected into C57BL/6J blastocytes using standard methods. In these cells, successful homologous recombination of the targeting construct results in expression of the *LacZ* gene encoding bacterial β-galactosidase under the control of the endogenous *Hgsnat* promoter from the targeted alleles (Fig. S1). Correct targeting was confirmed by Southern blot (data not shown) and by specific PCR that yielded different band patterns for wild-type (WT) (*Hgsnat^+/+^*), heterozygous (*Hgsnat^+/−^*) or homozygous (*Hgsnat^−/−^*) knockout mice (Fig. 1A). There was no indication of embryonic lethality and at birth HGSNAT-deficient mice were viable and completely undistinguishable from WT and heterozygous littermates. No statistical differences were detected in the body weight at any of the ages analyzed (Fig. S2).

No *Hgsnat* transcripts could be detected in the kidney of homozygous *Hgsnat^−/−^* male mice by northern blot analysis with a probe specific for murine *Hgsnat* (Fig. 1B). This observation was later confirmed by qRT-PCR in samples obtained from brain and liver (Fig. 1C). Accordingly, HGSNAT enzymatic activity was practically undetectable in brain and liver of *Hgsnat^−/−^* mice (Fig. 1D). Heterozygous mice showed reduced *Hgsnat* expression and HGSNAT activity (Fig. 1B-D). This lower than normal enzymatic activity in *Hgsnat^+/−^* mice did not alter the content of GAGs in tissues (data not shown), indicating that half of normal HGSNAT activity is enough to support normal catabolism of GAGs. Similar results were obtained in females (Fig. S5A,B). Taken together these results demonstrated efficient disruption of the *Hgsnat* gene.

### Study of *Hgsnat* gene expression in the CNS and somatic organs

The design of the targeting construct allows for analysis of the sites of endogenous *Hgsnat* expression through detection of β-galactosidase activity. *In toto* X-gal staining of the encephalon of *Hgsnat*^−/−^ mice showed widespread and strong blue signal of the reaction product throughout the forebrain and midbrain (Fig. S3, upper panel and A-C). In general, the pattern of expression was homogenous and diffuse, e.g. in the neocortex, the piriform lobe and thalamus (Fig. S3A-C), except for the hippocampus in which the signal was particularly intense in CA1 and CA3 cells, with lesser expression detected in the dentate gyrus (Fig. S3B). Lower but detectable levels of *LacZ* expression were observed in the cerebellum, with Purkinje cells evidencing the most intense signal in this structure (Fig. S3D). The widespread expression of the *Hgsnat* gene throughout the CNS was in agreement with the fact that the brain is the most affected organ in Sanfilippo syndrome type C (Kurihara et al., 1996; Martin et al., 1979).

We then evaluated *in toto* β-galactosidase activity in somatic organs (Fig. S4). The vast majority of organs analyzed showed expression of *Hgsnat.* In the kidney, the cortex stained more than the medulla; in the medullar region, positivity was associated with tubular structures that converged in the renal pelvis, namely papillary ducts (Fig. S4A). Very strong staining was observed in the ureter and, to a lesser degree, in the urinary bladder (Fig. S4A,B). In contrast, the lungs were practically negative, with minimal staining of the lobar bronchus (Fig. S4C). When the whole heart was incubated in X-gal, ventricles showed very weak and diffuse signal whereas atriums were strongly positive, and so was the pulmonary trunk (Fig. S4D). When the heart was sectioned to allow visualization of inner structures, a diffuse staining was observed in the inner surface of the myocardium of the left ventricle, particularly in the papillary muscles (Fig. S4E,F). In the transversal section of the atrium, the tricuspid valve stained strongly positive (Fig. S4G). The staining of the liver was moderately intense, but the gallbladder was markedly blue (Fig. S4H,I). The pancreas was faintly blue throughout (Fig. S4J). The parenchyma of the spleen was negative to X-gal staining, and the only structures that stained positive were identified as arterial blood vessels (Fig. S4K). Similarly, muscle fibers of the gastrocnemius muscle seemed negative for *LacZ* expression but muscular arterioles were markedly positive (Fig. S4L). When X-gal staining was performed on paraffin-embedded sections of the gastrocnemius muscle, a strong blue signal was observed in the arterial tunica media of these vessels (Fig. S4M). Finally, in the reproductive tract, in females the oviduct stood out as the most positive structure, although the follicles inside the ovary were also fairly positive (Fig. S4N); in testicles, the seminiferous tubules stained strongly with X-gal (Fig. S4O).

### CNS lysosomal pathology in HGSNAT-deficient mice

**Fig. 2.**
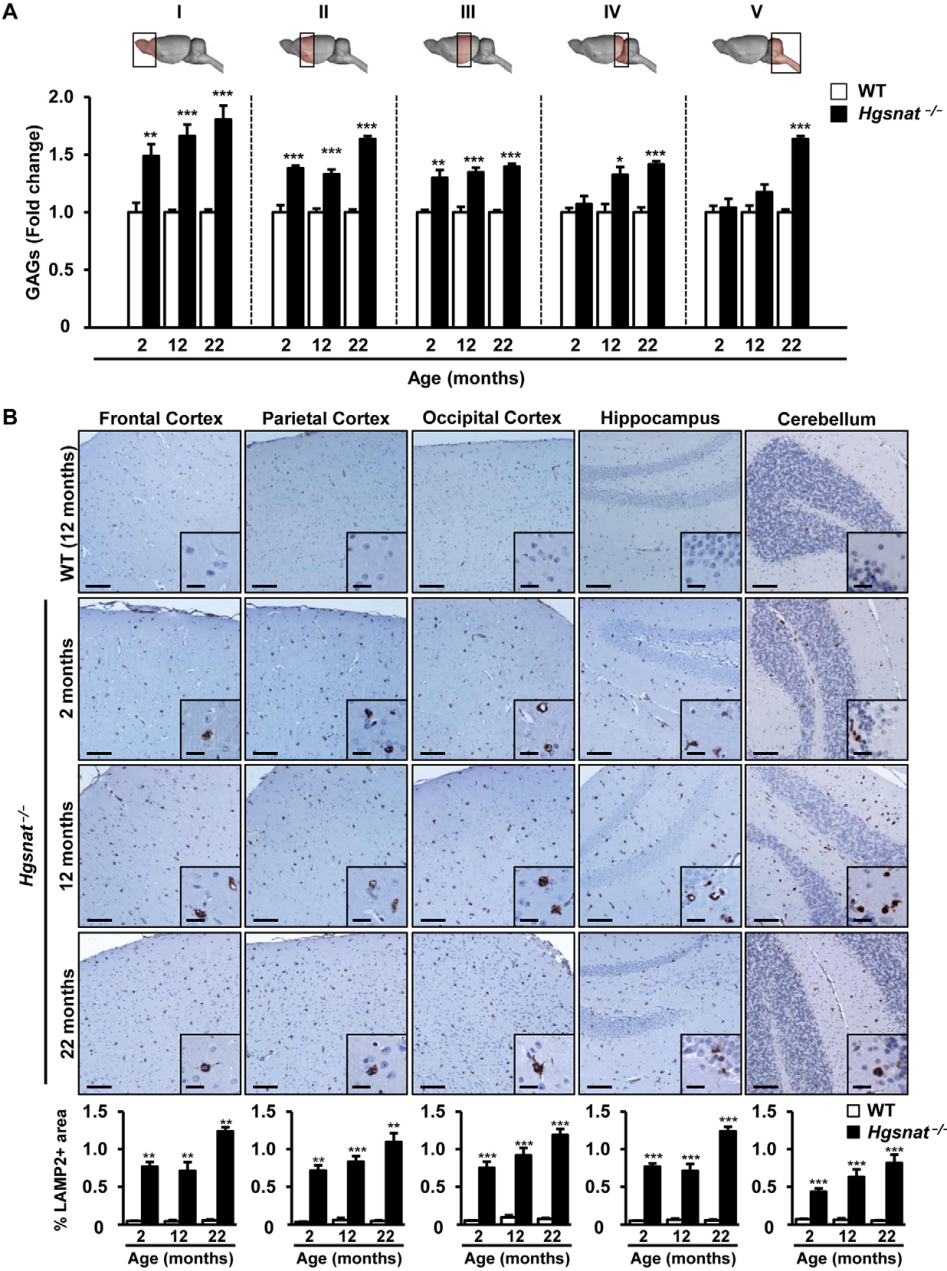
**CNS storage pathology in *Hgsnat*^−/−^ mice.** (A) GAG content in different brain regions (sections I-V illustrated in the diagrams above the plot) in WT and *Hgsnat*^−/−^ male mice at 2, 12 and 22 months of age; *n*=4-5 animals/group. Absolute values of CNS GAG content in WT animals are listed in Table S1. (B) Analysis of the lysosomal compartment by LAMP2 immunostaining in different brain regions of the same cohorts of animals as in A. HGSNAT-deficient animals showed a progressive increase in the size of the lysosomal compartment with age. Insets show a magnified region of the main panel. Histograms below the images depict the corresponding quantification of LAMP2+ signal for each brain region in each group; *n*=4-5 animals/group. Results are shown as mean±s.e.m.; *P<0.05, **P<0.01 and ***P<0.001 versus WT by two-tailed *t-*test for statistical comparison of HGSNAT-deficient mice with their respective controls. Scale bars: 100 µm; insets, 20 µm.

Neurological alterations are one of the main features of MPSIIIC disease (Héron et al., 2011; Malm and Månsson, 2010; Ruijter et al., 2008). Therefore, we set out to investigate the presence of lysosomal pathology in the brain of mice with HGSNAT deficiency. Animals of 2, 12 and 22 months of age were analyzed to evaluate the age of onset and the progression of the lysosomal pathology affecting the CNS. HGSNAT-deficient male and female mice showed a progressive increase in GAG accumulation in all brain regions analyzed, ranging from 4% to 80% over values observed in healthy littermates (Fig. 2A; Fig. S5C). In both sexes, the accumulation was particularly noticeable in the most frontal portions of the brain (Sections I-III; Fig. 2A; Fig. S5C), in which the excessive storage of GAG was readily detectable as early as 2 months of age. Consistent with the observed accumulation of GAGs, immunohistochemical analysis of brain sections with an antibody reactive to the lysosomal marker lysosomal associated membrane protein 2 (LAMP2) used as indicator of the size of the lysosomal compartment revealed a statistically significant increase in LAMP2+ area in all regions analyzed in the CNS of both *Hgsnat^−/−^* male and female mice (Fig. 2B; Fig. S5D). Furthermore, signal intensity increased with age in both sexes, suggesting the distension of the CNS lysosomal compartment worsened as animals grew.

**Fig. 3.**
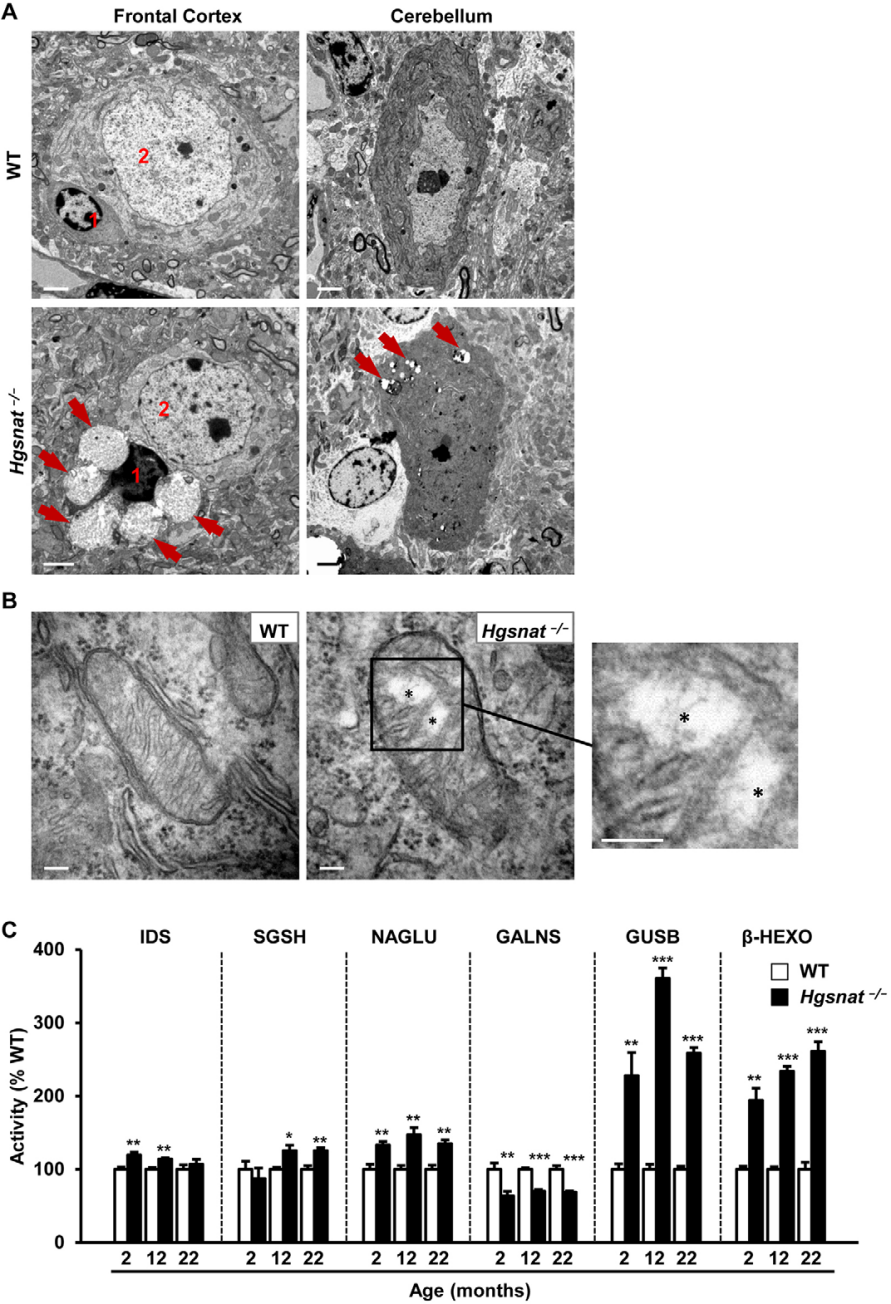
**Ultrastructural analysis and evaluation of lysosomal homeostasis in the CNS of HGSNAT-deficient mice.** (A) Transmission electron microscopy of samples from the frontal cortex (left panels) and cerebellum (right panels) of 5-month-old WT and *Hgsnat*^−/−^ male mice. Large electrolucent vacuoles (red arrows) were detected in perineuronal glial cells (1) associated with neurons (2) of the frontal cortex in HGSNAT-deficient male mice, whereas smaller vesicles with seemingly different content were observed in cerebellar Purkinje cells. (B) Representative images of mitochondria found in Purkinje cells of WT and *Hgsnat*^−/−^ mice. The mitochondrion from a *Hgsnat*^−/−^ Purkinje cell shows loss of normal mitochondrial structure and reduced cristae (asterisks). (C) Percentage of WT activity for a range of lysosomal enzymes analyzed in brain extracts from WT and *Hgsnat*^−/−^ male animals at 2, 12, and 22 months. *Hgsnat*^−/−^ mice showed altered activities of iduronate-2-sulfatase (IDS), N-sulphoglucosamine sulphohydrolase (SGSH), α-N-acetylglucosaminidase (NAGLU), N-acetylgalactosamine-6-sulfatase (GALNS), β-glucuronidase (GUSB) and β-hexosaminidase (β-HEXO); *n*=4-5 animals/group. Absolute values of CNS enzymatic activities in WT animals are listed in Table S2. Results are shown as mean±s.e.m.; *P<0.05, **P<0.01 and ***P<0.001 by two-tailed *t-*test for statistical comparison of HGSNAT-deficient mice with their respective controls. Scale bars: 2 µm in A, 100 µm in B.

This result was further confirmed by ultrastructural analysis of the encephalon by transmission electron microscopy, which showed an increase in the size of the lysosomal compartment in the cerebral cortex and cerebellum of HGSNAT-deficient mice (Fig. 3A). At 5 months of age, *Hgsnat^−/−^* animals but not WT littermates had large electrolucent vacuoles that seemed to be lysosomes loaded with GAGs in the cytoplasm of perineuronal glial cells apposed to neurons of the cerebral cortex (Fig. 3A, left panels). The juxtaposed neurons had few or no discernable storage lesions at this age (Fig. 3A, left panels). In the cerebellum, electrolucent vesicles of smaller size were detected in the cytoplasm of Purkinje cells in *Hgsnat^−/−^* mice (Fig. 3A, right panels). The content of these vesicles seemed different from that observed in cortical perineuronal glial cells (Fig. 3A, right panels). Furthermore, the cellular pathology observed in Purkinje cells from HGSNAT-deficient mice affected not only lysosomes but also mitochondria, which showed significant cristae loss and disorganization of their inner membrane system (Fig. 3B). Taken together, these data demonstrate the presence of progressive storage disease in the CNS of HGSNAT-deficient mice starting from an early age.

### Secondary lysosomal pathology in the CNS of HGSNAT-deficient mice

In LSDs, the loss of activity of a specific lysosomal enzyme results in the perturbation of normal lysosomal homeostasis, characterized, amongst other changes, by secondary alteration of other enzymatic activities and accumulation of additional compounds (Sardiello et al., 2009). In the brain of HGSNAT-deficient mice, we observed changes in the activities of several lysosomal enzymes, such as IDS, SGSH, NAGLU, GALNS, GUSB and β-HEXO (Fig. 3C; Fig. S6). These changes were detectable as early as 2 months of age and remained altered in older mice (Fig. 3C; Fig. S6).

### Neuroinflammation in the brain of mice deficient in HGSNAT

**Fig. 4.**
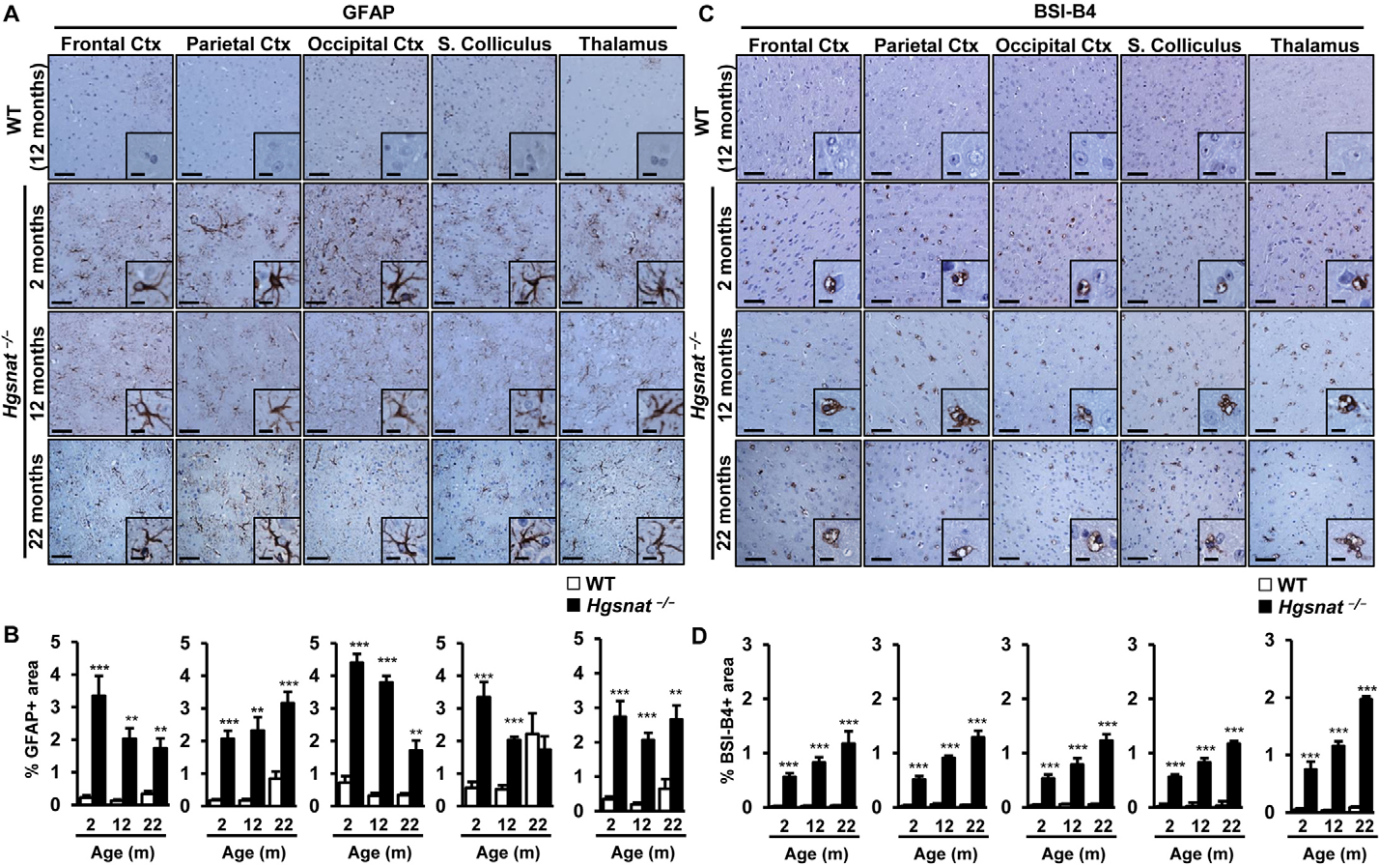
**Astrocytosis and progressive infiltration with activated microglia in the brain of *Hgsnat*^−/−^ mice.** (A) Representative images of the immunostaining of brain sections with an antibody specific for the astrocyte marker GFAP. WT and *Hgsnat*^−/−^ male mice were analyzed at 2, 12 and 22 months of age. Insets show a magnified region of the main panel. (B) Histograms represent the percentage GFAP+ area in each brain region analyzed; *n*=4-5 animals/group. (C) Representative images of the analysis of microgliosis by staining with BSI-B4 lectin in brain sections from the same cohorts. Microgliosis worsened with age in *Hgsnat*^−/−^ mice. (D) Histograms depict the quantification of BSI-B4+ area in each brain region analyzed; *n*=3-5 animals/group. Results are shown as mean±s.e.m.; **P<0.01, ***P<0.001 by two-tailed *t-*test for statistical comparison of HGSNAT-deficient mice with their respective controls. Scale bars: 50 µm; insets, 20 µm. Ctx, Cortex; S., Superior; m, months.

There is plenty of evidence from animal models (Martins et al., 2015; Ohmi et al., 2003; Wilkinson et al., 2012) and humans (Hamano et al., 2008; Kurihara et al., 1996; Tamagawa et al., 1985) indicating that there is activation of glial cells in all forms of Sanfilippo syndrome. To investigate if the deficiency in HGSNAT also caused a neuroinflammatory response in our animal model, we stained brain sections from 2-, 12- and 22-month-old mice with an anti-GFAP antibody that specifically recognizes glial fibrillary acidic protein expressed in activated astrocytes (Eng and Ghirnikar, 1994) and with the lectin BSI-B4, which under our staining protocol binds to activated microglia (Ribera et al., 2015; Streit and Kreutzberg, 1987). At the early age of 2 months, *Hgsnat^−/−^* mice of both sexes already showed marked astrocytosis in all brain regions analyzed (Fig. 4A,B; Fig. S7A,B). The increase in GFAP+ signal remained statistically significant at later time points in all regions, except for the superior colliculus in which significance was lost in very old animals (Fig. 4A,B; Fig. S7A,B). Similarly, BSI-B4 staining evidenced strong infiltration with activated microglia in the brains of *Hgsnat^−/−^* male and female mice, starting at 2 months and worsening with age (Fig. 4C,D; Fig. S7C,D). Quantification of signal intensity showed statistically significant increases in the BSI-B4+ area in *Hgsnat^−/−^* animals in all brain regions at all ages (Fig. 4C,D; Fig. S7C,D). Moreover, in the case of BSI-B4 staining, the progression with age was much more apparent than for GFAP staining. These results provide further evidence of progressive pathology in the CNS of HGSNAT-deficient mice.

### Loss of HGSNAT activity causes somatic pathology in mice

**Fig. 5.**
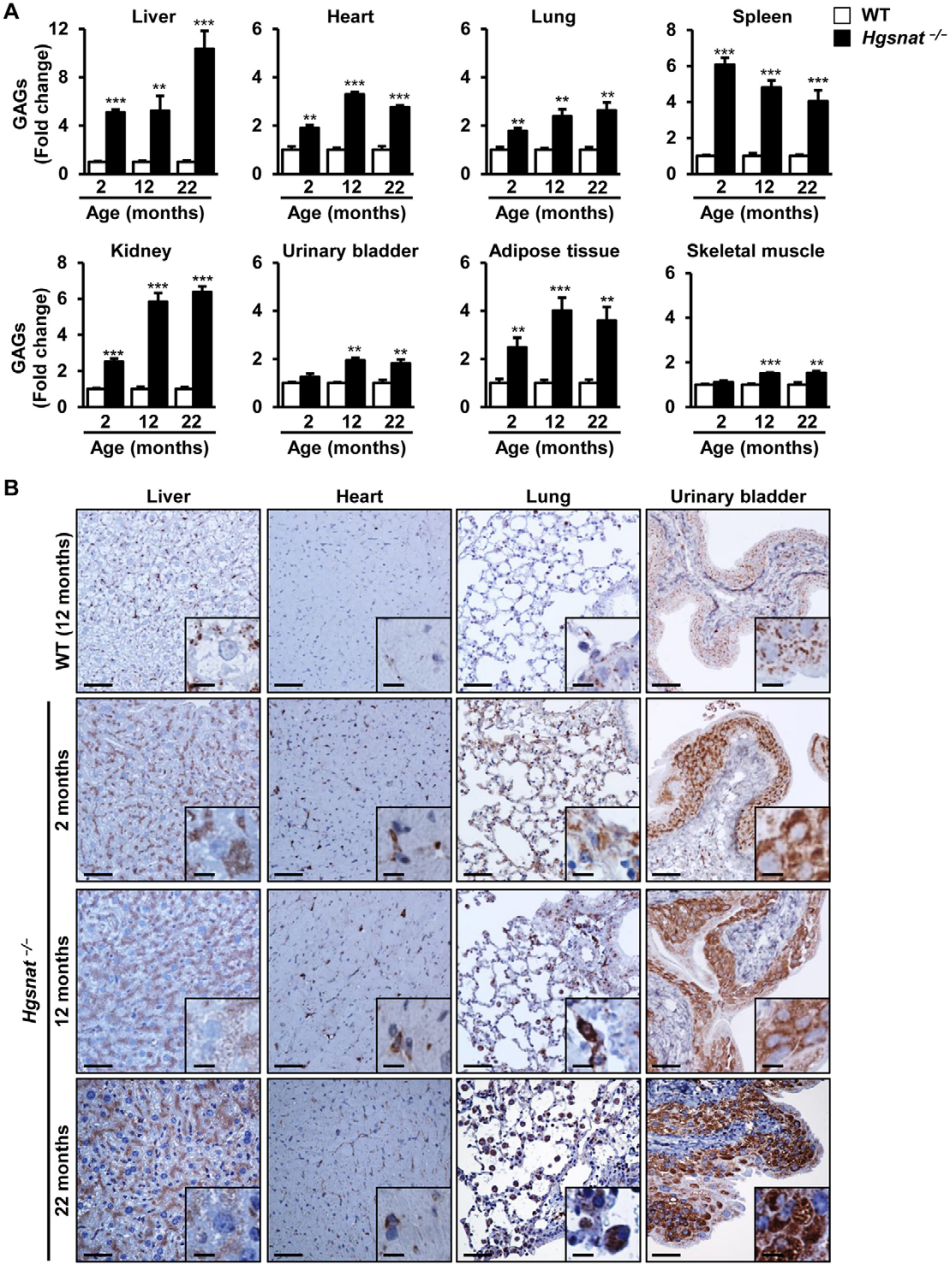
**Somatic lysosomal pathology in HGSNAT-deficient mice.** (A) Quantification of GAG content in somatic organs and tissues from healthy (WT) and *Hgsnat*^−/−^ male mice at 2, 12 and 22 months of age. Progressive GAG accumulation was observed in most somatic organs analyzed; *n*=4-5 animals/group. Absolute values of somatic tissue GAG content in WT animals are listed in Table S1. (B) Immunostaining against LAMP1 of liver, heart, lung and urinary bladder tissue sections demonstrating enlargement of the lysosomal compartment in affected male mice. Insets show a magnified region of the main panel. Results are shown as mean±s.e.m.; **P<0.01 and ***P<0.001 by two-tailed *t-*test for statistical comparison of HGSNAT-deficient mice with their respective controls. Scale bars: 50 µm; insets, 10 µm.

Lack of HGSNAT activity (Fig. 1D; Fig. S5B) led to the accumulation of GAGs in peripheral organs of male and female mice as early as 2 months of age (Fig. 5A; Fig. S8A). The build-up of undegraded GAGs was most noticeable in liver, spleen and kidney (Fig. 5A; Fig. S8A) and, in most organs, was progressive, showing a considerable increase particularly between 2 and 12 months of age (Fig. 5A; Fig. S8A). This excessive somatic accumulation of GAGs was accompanied by an expansion of the lysosomal compartment in male and female *Hgsnat*^−/−^ mice, as detected by immunostaining of liver, heart, lung and urinary bladder tissue sections with an antibody against the lysosomal marker lysosomal associated membrane protein 1 (LAMP1) (Fig. 5B; Fig. S8B). As observed for GAG content, in both sexes the size of the lysosomal compartment seemed to increase with age.

**Fig. 6.**
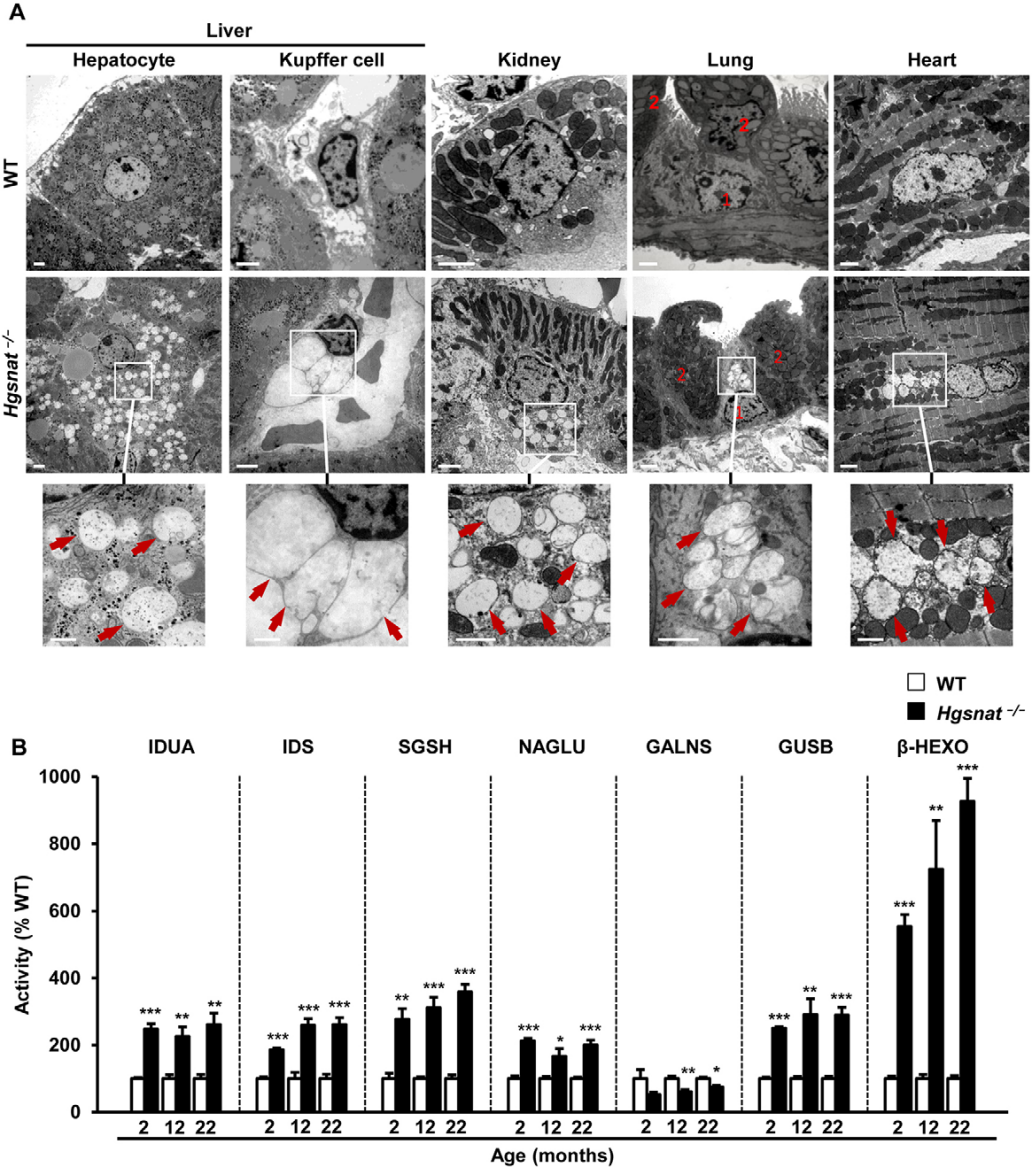
**Ultrastructural analysis and evaluation of lysosomal homeostasis in somatic organs of HGSNAT-deficient mice.** (A) Transmission electron microscopy of liver, kidney, lung and heart from WT and *Hgsnat*^−/−^ male mice at 5 months of age demonstrating the enlargement of the lysosomal compartment in different cells types. Electrolucent vacuoles (red arrows) of different size were detected in hepatocytes and Kupffer cells of the liver, in cells from the proximal tubules of the kidney, in ciliated cells of the bronchial tubes and in macrophage-like cells in the heart myocardium of *Hgsnat*^−/−^ mice. (1) Ciliated cells; (2) exocrine club cells. (B) Activity, as percentage of WT, of the lysosomal enzymes iduronidase (IDUA), iduronate-2-sulfatase (IDS), N-sulphoglucosamine sulphohydrolase (SGSH), α-N-acetylglucosaminidase (NAGLU), N-acetylgalactosamine-6-sulfatase (GALNS), β-glucuronidase (GUSB) and β-hexosaminidase (β-HEXO) analyzed in liver extracts of *Hgsnat*^−/−^ male mice at different ages; *n*=4-5 animals/group. Absolute values of liver enzymatic activities in WT animals are listed in Table S2. Results are shown as mean±s.e.m.; *P<0.05, **P<0.01 and ***P<0.001 by two-tailed *t-*test for statistical comparison of HGSNAT-deficient mice with their respective controls. Scale bars: 2 µm; insets, 1 µm.

At ultrastructural level, the pattern of enlargement of the lysosomal compartment in HGSNAT-deficient mice varied depending on the cell type and organ. In the liver, whereas hepatocytes showed a large number of small electrolucent vacuoles in their cytoplasm, Kupffer cells had few but very distended vesicles (Fig. 6A). Conversely, cells from the proximal tubules of the kidney showed vesicles of a size similar to those observed in hepatocytes (Fig. 6A). Ciliated cells from the bronchial tube of the lung and cells interspersed amongst myocardial cells of the cardiac ventricle had relatively large storage vesicles, which did not seem to be present in other cells of the parenchyma of these structures (Fig. 6A). Very similar observations were made in both sexes (Fig. S9). The excessive storage of undegraded substrates can give rise to organomegaly, a common feature of LSDs, including MPSIIIC (Bartsocas et al., 1979; Héron et al., 2011; Huh et al., 2013; Ruijter et al., 2008). *Hgsnat*^−/−^ mice did not show any organomegaly at 2 months of age, but organ weight increased with age to an evident hepato- and splenomegaly in older mice (Fig. S10A,B). Taken together, these results provide evidence of severe progressive storage pathology in peripheral organs of *Hgsnat*^−/−^ mice of both sexes.

As previously observed in the CNS, the lack of HGSNAT activity in liver of knockout mice resulted in secondary alteration of the activity of lysosomal enzymes involved not only in the degradation of GAGs (i.e. IDUA, IDS, SGSH, NAGLU, GALNS and GUSB) but also in the degradation of other macromolecules (i.e. β-HEXO), indicating a general alteration of normal lysosomal homeostasis (Fig. 6B; Fig. S10C). In most cases the activity of these enzymes was increased by several fold over the levels observed in healthy WT animals from an early age, and the increment was particularly noticeable for β-HEXO (Fig. 6B; Fig. S10C).

Finally, to assess general health status, we measured several biochemical parameters, including glucose, aspartate aminotransferase (AST), alanine aminotransferase (ALT), total bilirubin, creatinine and urea in sera obtained from 2-, 12- and 22-month-old animals. Of these, ALT and urea showed statistical differences from the values observed in WT animals (Fig. S10D), suggesting a mild degree of liver and kidney pathology. Taken together, these data demonstrate that *Hgsnat*^−/−^ mice have established disease in somatic organs as early as 2 months of age, with increased intracellular GAG storage, evident lysosomal distension and perturbation of normal lysosomal homeostasis, all of which could affect organ functionality.

### Behavioral deficits and shortened lifespan in *Hgsnat*^−/−^ mice

**Fig. 7.**
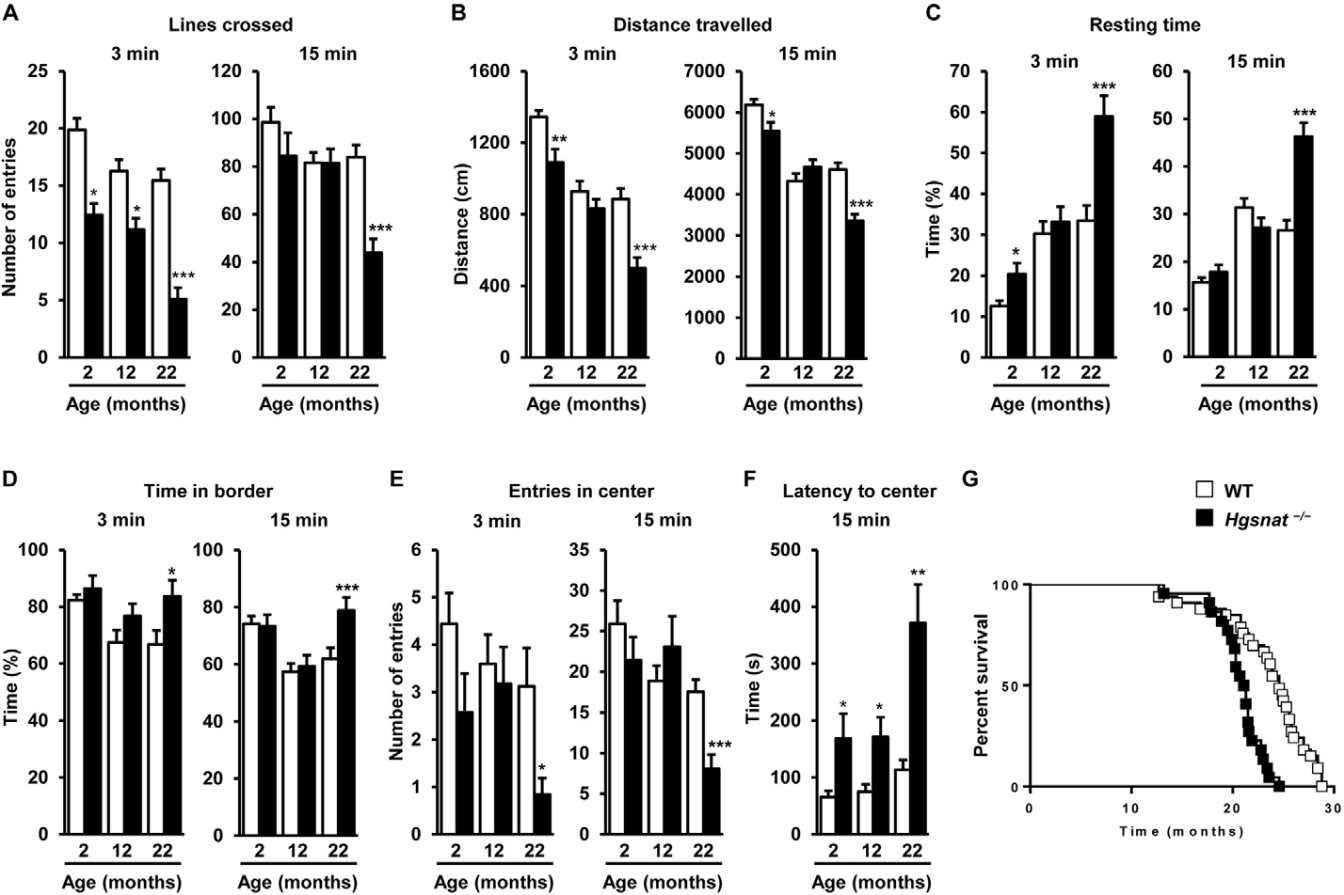
**Behavioral deficits and shorter survival of HGSNAT-deficient animals**. (A-F) The open field test was performed in naïve-tested WT and *Hgsnat*^−/−^ male mice at 2 (*n*=14-17), 12 (*n*=22-29) and 22 (*n*=16-19) months of age. Data correspond to the locomotor and exploratory activity recorded either during the first 3 min or a total of 15 min. Histograms depict the results of the parameters analyzed: (A) total number of lines crossed, (B) total distance travelled, (C) resting time, (D) time in border, (E) entries in center, and (F) latency to center. Results are represented as mean±s.e.m.; *P<0.05, **P<0.01 and ***P<0.001 by two-tailed *t-*test for statistical comparison of HGSNAT-deficient mice with their respective controls. (G) Kaplan–Meier survival analysis in WT (*n*=33) and *Hgsnat*^−/−^ (*n*=22) male mice. Median survival was 24.6 and 21.2 months, respectively. Kaplan–Meyer estimator followed by log-rank test, *P*=0.0001.

Behavioral disturbances are a hallmark of progressive neurological diseases such as MPSIIIC (Delgadillo et al., 2013; Héron et al., 2011; Malm and Månsson, 2010; Ruijter et al., 2008). To test if the behavior of *Hgsnat*^−/−^ mice was altered by a lack of HGSNAT, naïve *Hgsnat*^−/−^ males of 2, 12 and 22 months of age were subjected to the open field test, which evaluates the general locomotor and exploratory activity of mice in surroundings that are unknown to them (Bailey and Crawley, 2009). At all ages tested, *Hgsnat*^−/−^ mice crossed fewer lines and travelled less distance than WT littermates during the first three minutes of the recording period, which combined with the observation that *Hgsnat*^−/−^ mice spent more time resting, suggested hypoactive behavior (Fig. 7A-C). *Hgsnat*^−/−^ mice also took longer to reach the center of the arena, known to be most stressful zone, and did so on fewer occasions, spending more time at the border (Fig. 7D-F). Indeed, at 2 months of age, 28.5% of *Hgsnat*^−/−^ mice failed to enter the center of the arena in the first 3 min of recording, whilst all WT did. At 12 and 22 months the percentage of *Hgsnat*^−/−^ mice that took longer than 3 min to enter the center of the arena for the first time increased to 34% and 68.4%, respectively. Only one of the tested WT animals failed to visit the center during the first 3 min at 12 months of age, increasing to 25% in 22-month old animals. Taken together, these results suggest that *Hgsnat*^−/−^ mice suffer from anxiety. When the data corresponding to the recording over a period of 15 min was analyzed, a few of the statistical differences observed during the first 3 min at 2 and 12 months of age disappeared, including those that are more representative of locomotor activity, such as total resting time. This observation argues in favor of anxiety being a strong component of the behavior observed during the first 3 min of the test. In animals aged 22 months, the observations made at 15 min resembled those made after 3 min of recording, evidencing profound decline in motor functions at this age, which is compatible with late stage neurodegeneration. These behavioral alterations were in line with those previously observed by several other groups in two different mouse models of Sanfilippo disease (Fraldi et al., 2007; Haurigot et al., 2013; Hemsley and Hopwood, 2005; Lau et al., 2008; Li et al., 1999; McIntyre et al., 2010, 2014; Ribera et al., 2015).

Finally, the analysis of survival showed a highly statistically significant reduction in the lifespan of *Hgsnat*^−/−^ male mice, with a median survival of 21.2 months compared with 24.6 months for WT siblings (*P*=0.0001; Fig. 7G). Similarly, HGSNAT-deficient females were shorter-lived than WT counterparts (19.7 months and 23 months, respectively, *P*=0.0008; Fig. S11).

### MPSIIIC progresses more slowly than MPSIIIA and MPSIIIB in rodents

**Fig. 8.**
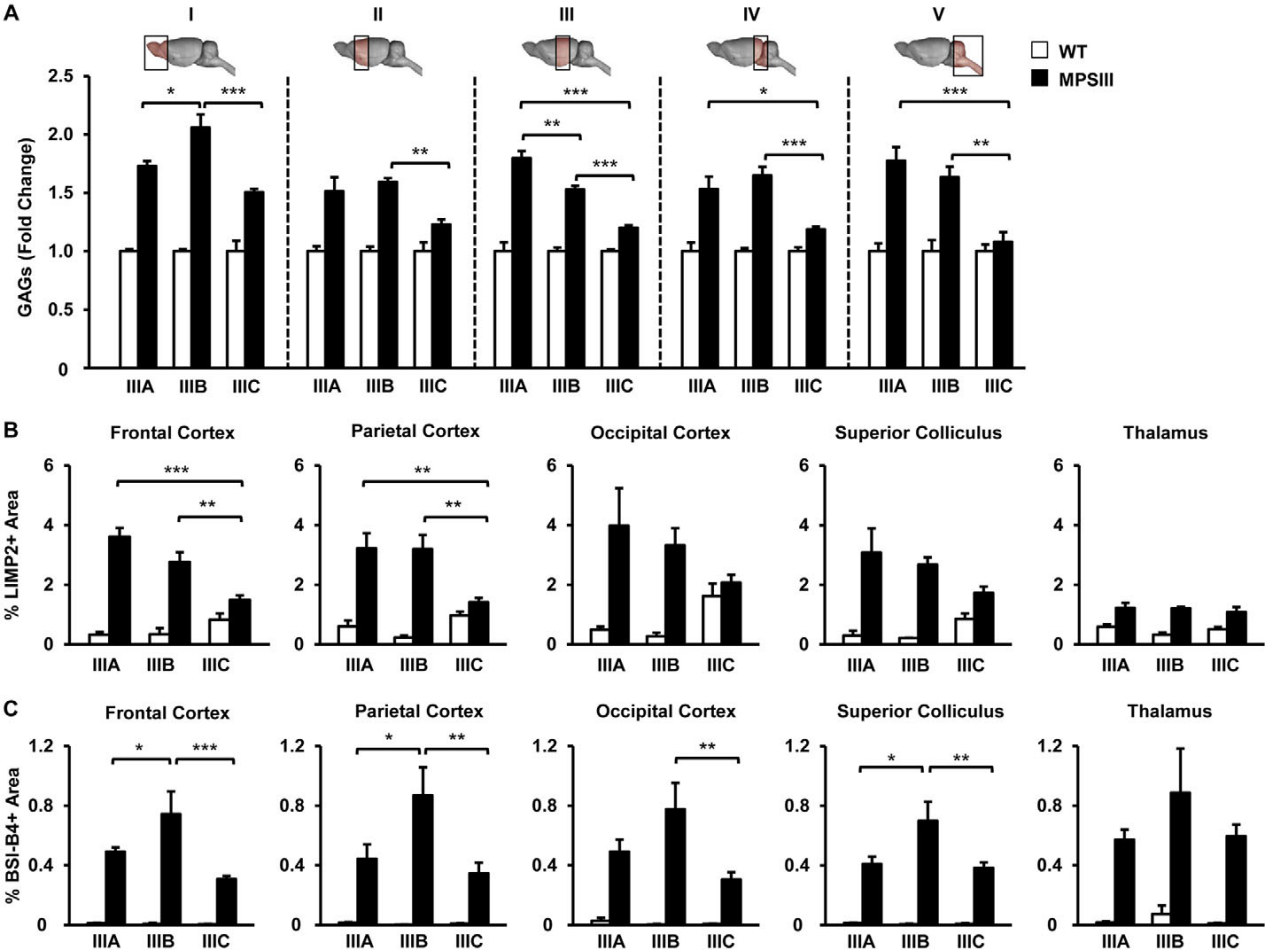
**Comparison of GAG accumulation, CNS lysosomal pathology and brain inflammation in different murine models of MPSIII disease.** (A) GAG content analyzed in different brain regions (sections I-V illustrated in the diagrams above the plot) from MPSIIIA, MPSIIIB and MPSIIIC mice at 5-6 months of age; *n*=4-5 animals/group. Absolute values of CNS GAG content in WT animals from different MPSIII colonies are listed in Table S3. (B) Analysis of the size of the lysosomal compartment through LIMP2 immunostaining and quantification of LIMP2+ area in different brain regions in the same cohorts of animals as in A; *n*=3-5 animals/group. (C) Analysis of microgliosis by staining with lectin BSI-B4 in brain tissue sections obtained from the same experimental groups. Histograms depict the quantification of positive area in each brain region analyzed; *n*=3-5 animals/group. Results are shown as mean±s.e.m.; *P<0.05, **P<0.01 and ***P<0.001 by one-way ANOVA followed by Tukey post-test.

HGSNAT-deficient males (MPSIIIC males) showed behavioral alterations that reproduced the observations we and others have previously reported in two other mouse models of Sanfilippo disease, MPSIIIA and MPSIIIB mice (Fu et al., 2007; Haurigot et al., 2013; Hemsley and Hopwood, 2005; Hemsley et al., 2009; Lau et al., 2008; Li et al., 1999; Ribera et al., 2015). The main difference between the MPSIIIC model and the MPSIIIA and MPSIIIB models was the progression of the behavioral disturbances; at 22 months of age the degree of behavioral changes in HGSNAT knockout mice was the same as in 6-month-old MPSIIIA or MPSIIIB mice (Haurigot et al., 2013; Ribera et al., 2015). Similarly, the lifespan of HGSNAT-deficient males and females was shortened but not as dramatically as that of MPSIIIA and MPSIIIB mice housed in the same animal facility (Haurigot et al., 2013; Ribera et al., 2015; Ruzo et al., 2012a,b). To investigate the reasons for the slower progression of neurological disease in the MPSIIIC model, we compared GAG content in different regions of the encephalon of MPSIIIA, MPSIIIB and MPSIIIC male mice of the same age (5-6 months) (Fig. 8A). In all regions analyzed, the accumulation of GAGs was less pronounced in MPSIIIC mice than in MPSIIIA or MPSIIIB mice, a decrease that reached statistical significance in most cases, particularly in the most caudal regions (Sections III, IV and V in Fig. 8A). In agreement with this, lysosomal-integrated membrane protein 2 (LIMP2) immunostaining indicated less expansion of the lysosomal compartment in MPSIIIC mice (Fig. 8B). Interestingly, this milder CNS storage disease observed in MPSIIIC mice reflected in less infiltration by activated microglia in the brain of these animals (Fig. 8C). This was particularly evident when MPSIIIC mice were compared with MPSIIIB mice. It should be noticed that whereas MPSIIIB and MPSIIIC animals are full knockouts, MPSIIIA mice arouse from a spontaneous point mutation and have a small percentage of residual activity (Bhattacharyya et al., 2001; Bhaumik et al., 1999; Haurigot et al., 2013; Ruzo et al., 2012a,b).

## DISCUSSION

Here we describe a novel animal model with targeted disruption of the *Hgsnat* gene. To generate this model we used ESC from the European Conditional Mouse Mutagenesis Program (EUCOMM) resource, which has extensively been used to generate hundreds of knockout mouse mutants for analysis of mammalian gene function (Hrabě de Angelis et al., 2015).

HGSNAT-deficient mice showed progressive accumulation of GAGs and expansion of the lysosomal compartment throughout the encephalon. At ultrastructural level, and in agreement with observations made in MPSIIIA and MPSIIIB models (Bhaumik et al., 1999; Haurigot et al., 2013; Jolly et al., 2007; Ribera et al., 2015; Ruzo et al., 2012a), cortical neurons showed minimal signs of storage whereas perineuronal glial cells juxtaposed to them had large intracytoplasmic vacuoles. Purkinje neurons showed vacuoles of smaller size in their somas, filled with material of different aspect from that observed in cortical perineuronal glial cells. Post-mortem analyzes of human and animal brains have shown secondary storage in LSDs; GM2 and GM3 gangliosides accumulate, for example, in MPSIII (Constantopoulos and Dekaban, 1978; Constantopoulos et al., 1980; Tamagawa et al., 1985). Storage of these compounds in cellular compartments different from those that accumulate GAGs, and sometimes even in different cells types (Jolly et al., 2007; Martins et al., 2015; McGlynn et al., 2004), could account for the dissimilar aspect of the storage vesicles found in glia and neurons. In addition, a proportion of the mitochondria of Purkinje cells showed structural abnormalities. The nature of mitochondrial alterations is not completely understood but a recent study has postulated that pathological GM storage in these organelles might be involved (Martins et al., 2015).

The changes in the activity of lysosomal hydrolases reflected the perturbation of normal lysosomal physiology by pathological storage of GAGs, in marked similarity to what we previously observed in MPSIIIB rodent brains (Ribera et al., 2015). This boost in lysosomal function is believed to be the consequence of the activation of the transcription factor EB (TFEB), a master regulator of lysosomal physiology, in an attempt to counteract pathological storage (Sardiello et al., 2009). In LSDs, TFEB translocates to the nucleus and binds to the coordinated lysosomal expression and regulation (CLEAR) element in the promoter of a number of genes that control lysosomal biogenesis and enzymatic activities (Ribera et al., 2015; Sardiello et al., 2009; Settembre et al., 2013). In contrast to this coordinated upregulation of lysosomal hydrolases, the activity of GALNS was decreased in the brain of HGSNAT-deficient mice, possibly resulting from direct inhibition by accumulated compounds. *In vitro* studies have suggested that GALNS is inhibited by HS (Rowan et al., 2013), and keratan sulfate, one of the substrates of GALNS, is increased in the serum of Sanfilippo animal models (Rowan et al., 2013) and humans with Sanfilippo syndrome (Tomatsu et al., 2005).

The brains of HGSNAT-deficient mice also showed astrocytosis and microgliosis from an early age. This glial response, a hallmark of human Sanfilippo syndrome (Hamano et al., 2008; Kurihara et al., 1996; Tamagawa et al., 1985), is believed to be at the epicenter of disease pathology. In particular, microgliosis, which can be detected as soon as 10 days after birth in MPSIII mice (Ausseil et al., 2008), seems to participate in neurodegeneration through multiple pathological mechanisms including, but not limited to, the secretion of cytokines and the production of reactive oxygen species (Archer et al., 2014; Ausseil et al., 2008; Hamano et al., 2008; Villani et al., 2007; Wilkinson et al., 2012).

At the somatic level, we found hepatomegaly and progressive accumulation of GAGs in all organs analyzed from HGSNAT-deficient mice, but most noticeably in liver, spleen and kidney as well as biochemical signs of modest liver and kidney damage. At all ages, GAG accumulation in the liver and spleen seemed to be slightly less pronounced in females, which otherwise showed very similar CNS and somatic phenotypes to HGSNAT-deficient males. The alteration of lysosomal hydrolases was even more pronounced in liver than in brain, but followed the same pattern. At the ultrastructural level, storage lesions seemed to affect specific cell types within each organ. For example, ciliated cells in the bronchus and cells interspersed in the myocardium had large quantities of cytoplasmic storage vesicles but club cells or cardiomyocytes did not.

HGSNAT-deficient males showed behavioral alterations characterized by anxiety in younger animals and hypoactivity in later stages of the disease. These observations reproduced those we and others have previously reported in MPSIIIA and MPSIIIB mice (Fu et al., 2007; Haurigot et al., 2013; Hemsley and Hopwood, 2005; Hemsley et al., 2009; Lau et al., 2008; Li et al., 1999; Ribera et al., 2015). The main difference between those models was the progression of the behavioral disturbances; at 22 months of age the degree of behavioral changes in HGSNAT knockout mice was the same as in 6-month-old MPSIIIA or MPSIIIB mice (Haurigot et al., 2013; Ribera et al., 2015), likely because of milder CNS storage disease observed in the former. GAG storage, the expansion of the lysosomal compartment and microgliosis were all less pronounced in the brains of adult MPSIIIC mice compared with age-matched animals from our MPSIIIA and MPSIIIB colonies. Similarly, the lifespan of HGSNAT-deficient males and females was shortened but not as dramatically as that of MPSIIIA and MPSIIIB mice housed in the same animal facility (Haurigot et al., 2013; Ribera et al., 2015; Ruzo et al., 2012a,b). These observations support the notion of slower progression of MPSIIIC in rodents when compared with MPSIIIA and MPSIIIB diseases, and are in agreement with the less aggressive clinical course attributed to MPSIIIC in humans (Héron et al., 2011; Malm and Månsson, 2010; Ruijter et al., 2008; Valstar et al., 2008; van de Kamp et al., 1981). In a study of a large cohort of French individuals affected by different forms of MPSIII, individuals with MPSIIIC were found to live significantly longer than individuals with MPSIIIA or MPSIIIB (Héron et al., 2011). Ruijter et al. (2008) estimated the median survival of Dutch individuals with MPSIIIC to be 34 years, ranging from 25 to 48, whereas the median age at death of individuals with MPSIIIA from Germany has been reported to be 15.2 years (range: 8.5-25.5 years) (Meyer et al., 2007). Indeed, there are several cases in the literature of individuals with MPSIIIC living into their fourth and fifth decades (Berger-Plantinga et al., 2004; Héron et al., 2011; Kurihara et al., 1996; Ruijter et al., 2008).

Interestingly, Martins et al. (2015) described hyperactivity and reduced anxiety in the open field test in another recently published mouse model of MPSIIIC, generated by the gene trap technology. There are important differences in the experimental procedures followed by each group that could account for this discrepancy. First, we followed the most standard open field protocol by which analysis is performed during the first few minutes of recording, before habituation to the environment occurs (Fraldi et al., 2007; Haurigot et al., 2013; Hemsley and Hopwood, 2005; Lau et al., 2008; Li et al., 1999; McIntyre et al., 2010, 2014; Ribera et al., 2015). Under these conditions, MPSIIIA and MPSIIIB animals have been described to be hypoactive (Fraldi et al., 2007; Haurigot et al., 2013; Hemsley and Hopwood, 2005; Lau et al., 2008; Li et al., 1999; McIntyre et al., 2010, 2014; Ribera et al., 2015), hyperactivity having been described only temporarily in very young (3-week-old) MPSIIIA mice that turn hypoactive by 6 weeks of age (Hemsley and Hopwood, 2005). Moreover, when we analyzed the behavior over a period of 15 min, a time at which habituation to the environment has already begun, younger HGSNAT-deficient mice showed identical behavior to WT animals, but older mice continued to show hypoactivity, indicating loss of motor functions. In contrast to this protocol, Martins et al. reported the total outcome after 60 min of recording, and this could have influenced the type of behavior evidenced by the test. Secondly, in their manuscript, Martins et al. only show the results of the open field test performed in females, in which statistical significance was reached at 6 and 8 month of age but disappeared as animals grew older (Martins et al., 2015). The authors stated that the same trend was observed in male mice, although in this case statistical difference was reached only at 8 months of age for most parameters. Conversely, we performed the open field test in HGSNAT-deficient males, as in our experience with different murine models of MPSIII the results obtained in females can vary considerably owing to the influence of cyclic sexual hormones. In both mixed and congenic MPSIIIA mouse strains, altered locomotor open field activity manifested mostly in male mice (Hemsley and Hopwood, 2005; Lau et al., 2008), an observation that authors attributed to the neuroprotective effect of estrogens (Hemsley and Hopwood, 2005). In addition, despite having almost identical genetic background, the mouse model described by Martins and colleagues (C57BL/6NCrl) showed a considerably reduced lifespan in comparison with ours (C57BL/6NTac), with most MPSIIIC-affected animals dead by the age of 70 weeks (∼14 months), further emphasizing the impact that differences in housing conditions, diet and experimental protocols can have on the phenotype described.

Two previous studies analyzed the profile of *HGSNAT* expression in human samples by northern blot (Fan et al., 2006; Hřebícˇek et al., 2006). Although both studies used the same commercial source of polyA+ RNA extracted from various human tissues, the results were somewhat different. Whereas Fan et al. (2006) found high levels of expression in heart, skeletal muscle, liver and kidney, with moderate expression in brain, small intestine, colon, spleen, and placenta and low levels in other organs, Hřebícˇek et al. (2006) reported that heart, leucocytes, lung, placenta and liver were the sites with the highest expression, with the lowest expression being observed in the brain. Taking advantage of the design of our targeting construct, in which the reporter bacterial protein β-galactosidase is expressed under the control of the endogenous *Hgsnat* promoter in the recombined allele, we performed a thorough anatomical description of the sites of *Hgsnat* expression in mice. *In toto* incubation of tissues and organs in X-gal solution revealed the encephalon as the organ most readily stained. Whereas coronal sections of the encephalon showed intense blue staining after only 4 h of incubation, most somatic organs needed an overnight incubation to become positive. This staining pattern is compatible with a disease in which the lack of enzymatic activity results mostly in neurological manifestations, but contradicts previous studies in WT mice in which the highest levels of HGSNAT activity were reported for spleen and lungs, and brain showed much lower activity – ∼sixfold lower than spleen – and in the same range as liver (Martins et al., 2015). Of note was the finding that splenocytes were negative for β-galactosidase activity and only blood vessels stained blue in the spleen. This was somewhat unexpected as splenomegaly is a frequent sign of Sanfilippo syndrome (Valstar et al., 2008, 2010a,b), and HGSNAT-deficient mice accumulated large amounts of GAGs in this organ (sixfold higher than WT). To a lesser degree, a similar observation was made in the liver. Given that hepatomegaly is not an uncommon finding in affected individuals (Buhrman et al., 2014; Cleary and Wraith, 1993; Héron et al., 2011; Huh et al., 2013; Ruijter et al., 2008; Valstar et al., 2008) we expected significant staining of this organ, almost in the same range as that observed in the brain. However, after an overnight incubation in X-gal solution there was only faint staining of hepatocytes and the only intensely blue structures were identified as blood vessels. Despite this weak *LacZ* expression in hepatocytes, the content of GAGs in the liver of HGSNAT-deficient animals was almost tenfold higher than in WT littermates, and at ultrastructural level, hepatocytes and, specially, Kupffer cells, showed a large number of electrolucent vesicles, an observation previously made in subjects affected by MPSIIIC (Kurihara et al., 1996). All tissues analyzed were cut across to ensure penetration of the X-gal reagent. Although technical limitations cannot be completely ruled out, these apparent discrepancies suggest intriguing pathophysiological mechanisms of disease. It might be the case that under normal circumstances the liver and spleen produce enough HGSNAT protein to cope with the normal turnover of GAGs in these tissues. However, in a disease state these organs might participate in the clearance of GAGs from the circulation, as GAG levels are considerably increased in the blood and urine from individuals with all forms of MPS (de Ruijter et al., 2012, 2013; Pievani et al., 2015; Rowan et al., 2013; Sifuentes et al., 2007; Tomatsu et al., 2005). That macrophages might buffer storage overload by uptake of GAGs from the interstitial space or blood was proposed a few years ago to justify the presence of large storage vesicles in glial cells juxtaposed to neurons with very little evidence of storage disease, or the presence of foamy macrophages in almost all tissues of MPS animal models (Jolly et al., 2007). Alternatively, the rate of turnover of GAGs in liver and spleen might be higher than in other tissues, hence the lack of enzymatic activity has a much bigger impact in terms of substrate accumulation. Finally, another plausible explanation to such a pronounced increase in GAG content in organs that do not seem to express *Hgsnat* at high levels could be that the accumulation of HS and derivatives causes inhibition of lysosomal enzymes involved in the degradation of other GAGs. In other words, the GAG quantified by the unspecific Blyscan method is a GAG different from HS. As mentioned before, a recent report suggested that in Sanfilippo syndrome, the enzyme GALNS might be inhibited by HS and heparin (Rowan et al., 2013) resulting in secondary accumulation of keratan sulfate in humans (Tomatsu et al., 2005). Supporting this hypothesis we found decreased activity of GALNS in the liver of HGSNAT-deficient mice.

In summary, the disruption of the *Hgsnat* gene in mice led to appearance of a wide range of signs of CNS and somatic pathology that recapitulate human MPSIIIC at biochemical, histological and functional level (Delgadillo et al., 2013; Héron et al., 2011; Huh et al., 2013; Kurihara et al., 1996; Malm and Månsson, 2010; Martin et al., 1979; Turki et al., 1989). This new animal model should prove a valuable tool for the study of the pathophysiology of the disease as well as for the development of novel therapeutic approaches. Although mice fully deficient in HGSNAT represent the most severe phenotype of MPSIIIC disease, any evidence of therapeutic efficacy demonstrated in this model would be encouraging for clinical translation to human individuals with a broader clinical spectrum.

## MATERIALS AND METHODS

### Animals

C57BL/6N-A/a ESC clones carrying a reporter (*LacZ* gene) tagged insertion in the murine *Hgsnat* gene [*Hgsnat^tm1a(EUCOMM)Wtsi^*] available through the International Mouse Phenotyping Consortium (IMPC, www.mousephenotype.org) were obtained. The clone EPD0485_4_B08 was microinjected in C57BL/6J blastocytes in the Transgenic Animal Unit of the Center of Animal Biotechnology and Gene Therapy (CBATEG) at Universitat Autònoma de Barcelona (UAB), and the resulting male chimeras were bred with C57BL/6NTac females to generate *Hgsnat* knockout offspring, which were 100% C57BL/6NTac. Genotyping was performed by PCR analysis using both locus-specific and *LacZ* cassette-specific primers *Hgsnat*-Fw: 5′-ACA AAT ACC TTG TTC CAT TCC GCC A-3′; Internal-Rev: 5′-GCC ACC CAA CTG ACC TTG GGC-3′; *Hgsnat*-Rev: 5′-ACT GCA CCT CTG CTC CAG TTA GA-3′. Bands of 395 or 329 bp were obtained for WT or knockout alleles, respectively. Homozygous mutant mice (*Hgsnat^−/−^* mice) were obtained by mating heterozygous littermates. Mice were fed *ad libitum* with standard diet (Harlan-Teklad) and maintained under a light-dark cycle of 12 h. All experimental procedures were approved by the Ethics Committee for Animal and Human Experimentation of UAB.

### Sample collection

Mice were anesthetized (100 mg/kg ketamine and 10 mg/kg xylazine), blood was extracted by cardiac puncture, and animals were intracardially perfused with 12 ml PBS to clear remaining blood from tissues. Brain and somatic tissues were collected and kept at −80°C or fixed in formalin. For the analysis of *LacZ* expression, animals were anticoagulated by intraperitoneal injection of 500 IU of heparin (Hospira) prior to anesthesia.

### RNA analysis

For northern blot analysis, total RNA was obtained from kidneys homogenized in Tripure Isolation Reagent (Roche) and purified following manufacturer's instructions. 20 μg of RNA were electrophoresed in 1% denaturing agarose gels, transferred to nylon membranes, and blots hybridized with ^32^P-labeled *Hgsnat* cDNA probe labeled by random oligopriming (GE Healthcare). For qRT-PCR analysis, total RNA from brain or liver was purified using RNeasy Mini Kit (Qiagen), and 1 μg was retrotranscribed with Transcriptor First Strand cDNA Synthesis Kit (Roche). Quantitative PCR was performed in a Light Cycler^®^ 480 (Roche) using Light Cycler^®^ 480 SybrGreen I Master (Roche) and primers specific for *Hgsnat* (forward: 5′-CGG CGT TCT TCT GCG AAC CG-3′; reverse: 5′-GGT CGG CCA CAG TCA GTC CG-3′) and normalized to the expression of mouse *Rplp0* gene (forward: 5′-GGC CCT GCA CTC TCG CTT T-3′; reverse: 5′-TGC CAG GAC GCG CTT GT-3′).

### X-gal staining

Tissues were dissected and fixed in 4% paraformaldehyde (Sigma) for 1.5 h and incubated protected from light at 37°C in X-gal (5-bromo-4-chloro-3-indoyl-β-d-galactopyranoside) solution [0.4 mg/ml X-gal (Sigma), 200 mM potassium ferrocyanide, 200 mM potassium ferrocyanate, 1 M MgCl_2_ diluted in PBS]. Coronal sections of the encephalon were incubated for 4 h, whereas sagittal sections and somatic tissues were incubated overnight. To stop the reaction, X-gal solution was removed and tissues were washed twice with PBS. Samples were kept in 10% neutral buffered formalin at 4°C until images were captured under a SMZ1000 zoom stereomicroscope (Nikon).

### Activity of lysosomal enzymes

Brain and liver samples were sonicated in 250 and 500 µl of water, respectively. Homogenized tissues were clarified by centrifugation and supernatants were recovered to assay enzyme activities using 4-methylumbelliferone-derived ﬂuorogenic substrates based on standard protocols (Hopwood et al., 1979; Ribera et al., 2015; Voznyi et al., 1993; Zhao et al., 1991). HGSNAT activity was assayed in 90 µg of protein extracts incubated with 3 mM 4-methylumbelliferyl-β-D-glucosamine (Moscerdam Substrates) supplemented with 12 mM acetyl coenzyme A (Sigma) for 3 h at 37°C. Iduronidase (IDUA) activity was assayed in 15 µg of tissue protein extracts incubated for 1 h at 37°C with 4-methylumbelliferyl α-L-iduronide 2 mM (Glycosynth) at pH 3.5. For iduronate-2-sulfatase (IDS) activity quantification, 15 µg of total protein were incubated with 1.25 mM 4-methylumbelliferyl-α-L-iduronide-2-sulfate (Moscerdam Substrates) for 4 h at 37°C followed by a second incubation with a pool of lysosomal enzymes from bovine testis (LEBT-M2, Moscerdam Substrates) for 24 h at 37°C. N-sulfoglucosamine sulfohidrolase (SGSH) and N-acetylgalactosamine-sulfate (NAGLU) activities were analyzed as previously described (Haurigot et al., 2013; Ribera et al., 2015). GALNS activity was assayed in 10 µg of total protein with a first incubation step with 10 mM 4-methylumbelliferyl β-D-galactopyranoside-6-sulfate (Toronto Research Chemicals) for 17 h at 37°C followed by a second incubation with β-galactosidase (Sigma) for 2 h at 37°C. β-hexosaminidase (β-HEXO) was assayed in 0.4 µg of protein at pH 4.5 for 1 h at 37°C with 5 mM 4-methylumbelliferyl N-acetyl-β-D-glucosaminide (Sigma). For α-N-acetylglucosaminidase (GUSB), activity was assayed in 15 µg (brain) or 5 µg (liver) of protein incubated for 1 h with 2 mM 4-methylumbelliferyl-β-D-glucuronide (Sigma) at pH 4.8 and 37°C. After stopping each enzymatic reaction by increasing the pH, the released fluorescence was measured with a FLx800 fluorimeter (BioTek Instruments). Results were calculated as nanomoles of substrate cleaved per hour per mg of protein quantified by Bradford assay (Bio-Rad), and then presented as a percentage of the activity of the WT, which was set to 100%.

### GAG quantification

Tissues were weighed and digested overnight in 0.2 mg/ml proteinase K solution. Extracts were centrifuged and supernatants were clarified by filtration in 0.22 μm microporous membrane-containing filters (Ultrafree MC, Millipore). GAG content was determined using the Blyscan sulfated glycosaminoglycan kit (Biocolor) using chondroitin 4-sulfate as standard and was normalized to wet tissue weight. Owing to the inherent technical variability of the assay, the GAG content of WT animals at each age was set to 1 to allow comparison of data obtained at different points in time.

### Histology

Formalin-fixed, paraffin-embedded tissue sections were incubated overnight at 4°C with rat anti-LAMP1 (SC-19992, Santa Cruz Biotechnology, 1:100), rat anti-LAMP2 (ab13524, Abcam, 1:500), rabbit anti-LIMP2 (NB400; Novus Biologicals, 1:100), rabbit anti-GFAP (Z0334, Dako Cytomation, 1:1000) and BSI-B4 lectin (L5391, Sigma, 1:100). For bright-field immunostaining, biotinylated rabbit anti-rat IgG (E0467, Dako, 1:300) and biotinylated goat anti-rabbit IgG (31820, Vector Laboratories, 1:300) were used as secondary antibodies. Bright-field sections were stained with 3,3-diaminobenzidine (Sigma) and counterstained with haematoxylin. Images were obtained with an Eclipse 90i optical microscope (Nikon). LIMP2, LAMP2, GFAP and BSI-B4 signals were quantified with the NIS-Elements Advanced Research 2.20 software (Nikon) in 3-5 20× images of each brain region of each animal using the same signal threshold settings for all images. The percentage positive area of each image was then calculated.

### Electron microscopy

Frontal cortex, cerebellum, liver, lung, heart and kidney were processed for transmission electron microscopy as previously described (Ruzo et al., 2012a) and visualized with a H-7000 transmission electron microscope (Hitachi).

### Open field test

Animals were always analyzed between 9:00 A.M. and 2:00 P.M., to minimize influence of circadian cycles. After placing the mouse in the corner of a brightly lit chamber (41×41×30 cm) divided in three squared concentric regions (center, 14×14 cm; periphery, 27×27 cm; and border, 41×41 cm), movements were recorded with a video-tracking system (SmartJunior v3, Panlab). Motor and exploratory activities were evaluated during the first three minutes, except for latency to center, which was recorded over 15 min, as several MPSIIIC mice failed to enter the arena during the first 3 min of the test.

### Metabolite analysis

ALT, AST, total bilirubin, creatinine, urea and glucose were measured in serum samples by spectrophotometry with a Cobas Mira Analyzer (Roche) by the Veterinary Clinical Biochemistry Lab of the Veterinary School at UAB.

### Statistical analysis

Results are expressed as mean±s.e.m. Statistical comparisons were made with either unpaired *t*-test two-tailed or one-way analysis of variance (ANOVA). Multiple comparisons were made using Dunnett or Tukey post-tests. Statistical significance was considered if *P*<0.05. The Kaplan–Meier estimate was applied to survival analysis, followed by log-rank test for comparisons.
